# Attenuating Pain With the Past: Nostalgia Reduces Physical Pain

**DOI:** 10.3389/fpsyg.2020.572881

**Published:** 2020-10-13

**Authors:** Mike Kersten, Julie A. Swets, Cathy R. Cox, Takashi Kusumi, Kazushi Nishihata, Tomoya Watanabe

**Affiliations:** ^1^Department of Psychology, University of Idaho, Moscow, ID, United States; ^2^Department of Psychology, Texas Christian University, Fort Worth, TX, United States; ^3^Graduate School of Education, Kyoto University, Kyoto, Japan

**Keywords:** nostalgia, physical pain, emotion, health, pain tolerance

## Abstract

Previous work has found that nostalgia, a sentimental longing for the past, is associated with psychological, emotional, and social benefits. Recent research has demonstrated that nostalgic reflection also can improve individuals’ physical health (i.e., exercise) and reduce temperature-related pain. Building on this, two experiments examined how nostalgia can reduce people’s pain perceptions (i.e., reduced severity and increased tolerance). Specifically, Study 1 showed that inducing nostalgia through a writing task decreased perceived pain severity (i.e., intensity) among self-reported chronic pain sufferers. Study 2, in turn, demonstrated that Japanese individuals experienced increased pain tolerance (i.e., the maximum level of pain a person can tolerate) for a pressure algometer task following thoughts of nostalgia (vs. a control prime). This work provides evidence that nostalgic reflection may serve as a psychological resource to reduce the perceived severity of physical pain.

## Introduction

Pain is a pervasive problem, affecting individuals globally. In the United States, one in five adults, or 50 million Americans, suffer from chronic pain (i.e., pain persisting longer than 3 months; Dahlhamer et al., 2018). Pain is often considered a silent epidemic because of (a) a lack of public awareness about its prevalence and (b) the likelihood of pain increasing physical, psychological, social, and financial costs. Research has shown that chronic pain leads to reduced health and quality of life, greater relationship conflict, and higher rates of depression and suicide (e.g., Takai et al., 2015).

The most common form of pain treatment is pharmacological therapy. Despite the integral role that medication plays in pain management, research suggests not all individuals experience the same degree of relief from prescription remedies. Breivik et al. (2006) found that nearly half of chronic sufferers do not use medication to treat their pain, with many persons citing drug-related complications (e.g., side effects and addiction) as the reason for their discontinued use. As a result, approximately two-thirds of chronic pain sufferers report implementing forms of non-drug treatments (Breivik et al., 2006).

Several interdisciplinary practices, such as mindfulness (e.g., Tai Chi), guided imagery, relaxation, and biofeedback, modulate pain perception by increasing resilience (Villemure and Bushnell, 2002; Roditi and Robinson, 2011; Peng, 2012). Cognitive-behavioral practices, such as reframing and decatastrophizing, can also help to alleviate pain (Gatchel et al., 2007). Collectively, these types of interventions are effective for chronic pain patients, who see improved physical and psychological health compared to control conditions (Ehde et al., 2014).

However, there are problems with the feasibility of these options. First, many methods necessitate long-term commitment (i.e., time, dedication) to be effective. Second, persons who engage in mind-body exercises (e.g., yoga) are mostly higher socioeconomic groups (e.g., college-educated, urban dwellers; Cramer et al., 2016). Finally, strategies grounded in physical exercise may be difficult for chronic sufferers given their limited mobility and/or pain experience, making different body postures difficult to perform (Peng, 2012). This is especially true for older adults (Morone and Greco, 2007). One strategy that may be effective in reducing pain perceptions is the psychological resource of nostalgia: a sentimental longing for the past.

Although nostalgia can be a bittersweet experience infused with sentiments of negativity (e.g., separation from a loved one; Sedikides et al. (2008), coded narratives of nostalgic events often contain greater traces of positive (e.g., happiness and gratitude) rather than negative emotion (e.g., loneliness, depression; Hepper et al. (2012). This has led some researchers to suggest that nostalgia helps people manage unpleasant or harmful psychological states. For example, nostalgia’s role in regulating threats to well-being has been shown to counteract feelings of loneliness, meaninglessness, and boredom (see e.g., Sedikides and Wildschut, 2020).

One question is whether nostalgia can help individuals when they experience *physical* harm, specifically, physical pain. Research by Zhou et al. (2012) began to address this question. These studies showed that participants in a state of nostalgia were able to hold their hand in a bucket of ice water for a longer period of time compared to participants asked to think about an ordinary event. In addition, uncomfortably low temperatures, both experimentally induced and naturally occurring, were associated with greater frequency of nostalgic thought. What is not answered by this work, however, is whether nostalgia can (a) regulate pain more generally, not necessarily associated with temperature; (b) be functional for individuals who suffer from chronic pain; and (c) impact subjective self-reports of pain severity.

The current research examined whether nostalgic thinking can decrease pain. There are qualitative differences in defining pain *severity* (i.e., intensity) and *tolerance* (i.e., the maximum level of pain a person can tolerate). Although both fall under the broader category of *pain perception*, the two constructs measure different experiences. For both studies here, we use *pain perception* to discuss general study findings; however, each experiment uses specific terminology as intended by scale/instrument developers. Study 1 was conducted among a community sample of chronic pain sufferers. Study 2 examined the extent to which nostalgia increased tolerance for mechanically evoked pain via a pressure algometer. It was hypothesized, in both experiments, that activating thoughts of nostalgia would lead to reduced pain [i.e., lower self-reported severity (Study 1); higher tolerance to evoked pain (Study 2)]. Given that nostalgia research is predominantly conducted among White/Caucasian samples, which is also reflected in our first experiment, an additional goal of the second study was to test the cultural generalizability of nostalgia in a sample of Japanese individuals.

## Study 1

Study 1 tested whether recalling a nostalgic (vs. ordinary) event would lead to lower perceived pain severity among chronic sufferers.

### Method

#### Participants

Power was determined based on the moderate (*d* = 0.38) to large (*d* = 0.81) effect sizes found in the nostalgia literature (Ismail et al., 2020). Using G^∗^Power (Faul et al., 2007) for a mixed-design analysis of variance (ANOVA) to detect a moderate effect (*f* = 0.25) with power set at 0.80, a minimum of 34 individuals were needed. We recruited 206 workers from Amazon’s Mechanical Turk (MTurk; 123 females; age range 20–74 years, *M*_*age*_ = 37.31, *SD*_*age*_ = 11.24; 163 White/Caucasian).

To select chronic pain sufferers, we presented participants with a modified version of the Standards of Chronic Pain scale (Deyo et al., 2014). Specifically, individuals rated (a) the length of time that their pain has been an ongoing problem (*Less than 1 month*; *1–3 months; 3–6 months*; *6 months-1 year; 1–5 years; More than 5 years)* and (b) how often their pain had been an ongoing problem over the past 6 months (*Every day or nearly every day in the past 6 months; At least half the days in the past 6 months; Less than half the days in the past 6 months*). Participants met the criteria for having chronic pain if they provided a response of *greater than 3 months* to Question A and a response of *at least half the days in the past 6 months* to Question B. Only these individuals were eligible to participate in the study and were paid $2.00.

#### Materials and Procedure

Study 1 was conducted online. Following a baseline measure of pain severity, persons were randomly assigned to write about a nostalgic or ordinary (control) event. Participants then completed a post-manipulation measure of their perceived level of pain and provided demographic information. The study took approximately 15 min to complete.

##### Time 1 pain perception

Participants completed the Pain Numeric Rating Scale (NRS-11; Hartrick et al., 2003), which is a widely used clinical assessment of physical pain. Individuals rated their current pain level (*How would you rate your pain right now?*) on an 11-point scale (0 = *No pain*; 10 = *Worst pain imaginable*). Three additional items were included, but not analyzed, given the state nature of the nostalgia manipulation: *How would you rate your USUAL level of pain during the last week?*; *How would you rate your LOWEST level of pain during the last week?*; and *How would you rate your WORST level of pain during the last week?*

##### Nostalgia manipulation

Individuals were randomly assigned to either a nostalgia or control condition (see e.g., Wildschut et al., 2006). In the nostalgia condition, participants were asked to *think of a nostalgic event in your life*… *specifically, try to think of a past event that makes you feel most nostalgic*. Participants in the control condition were instructed to *bring to mind an ordinary event in your life*. Following this, everyone completed a manipulation check by answering three questions (see e.g., Routledge et al., 2008): *Right now, I am feeling quite nostalgic*; *Right now, I am having feelings of nostalgia*; and *I feel nostalgic at this moment*. Responses were made on a 9-point scale (1 = *Strongly disagree*, 9 = *Strongly agree*; α = 0.99).

##### Time 2 pain perception

Participants were again asked to report their level of *current* perceived pain severity using the NRS.

### Results and Discussion

#### Manipulation Check

Nostalgic participants (*M* = 7.49, *SD* = 1.64) reported greater state-level nostalgia than did those who wrote about an ordinary event (*M* = 3.79, *SD* = 2.35), *F*(1,204) = 173.48, *p* ≤ 0.001, *d* = 1.85.

#### Pain Perceptions

A 2 (between-subjects variable: nostalgia vs. ordinary event) × 2 (within-subjects variable: Time 1 vs. Time 2) mixed-design analysis of variance (ANOVA) was performed on pain perception scores. The results revealed a non-significant main effect of nostalgia manipulation, *F*(1,204) = 2.62, *p* = 0.107, *d* = 0.36, and a significant main effect of time, *F*(1,204) = 9.84, *p* = 0.002, *d* = 0.88. There was also a significant nostalgia x time interaction, *F*(1,204) = 7.67, *p* = 0.006, *d* = 0.79 (see Table 1). Simple main effects revealed that participants in the nostalgia condition reported lower pain severity at Time 2 compared to Time 1, *F*(1,204) = 17.79, *p* ≤ 0.001, *d* = 0.99. There was no effect of time for individuals who recalled an ordinary event, *F*(1,204) = 0.07, *p* = 0.797, *d* = 0.06. Looked at differently, there was no difference between nostalgia and control conditions at Time 1, *F*(1,204) = 0.55, *p* = 0.459, *d* = 0.11. At Time 2, however, participants who recalled a nostalgic event had lower perceived pain severity compared to individuals who recalled an ordinary event, *F*(1,204) = 5.50, *p* = 0.020, *d* = 0.65.

**TABLE 1:** Pain severity scores as a function of nostalgia condition.

	Time 1		Time 2	
	*M*	*SD*	*M*	*SD*
Nostalgia Condition	5.63	1.95	5.15	1.92
Control Condition	5.83	1.98	5.80	2.06

*Higher scores indicate greater perceived pain (Study 1).*

Study 1 offers initial support for the physical pain-buffering effects of nostalgic reverie. Although no differences in perceived pain severity emerged between the nostalgia and control conditions at baseline, chronic sufferers reported their physical pain to be less severe after recalling a nostalgic (vs. ordinary) event. These findings indicate that, like other psychological resources that help reduce chronic pain (e.g., mindfulness and meditation), nostalgia may provide a form of perceived pain relief.

There are some limitations to be addressed. Study 1 was focused on subjective ratings of perceived pain severity, but the real levels of pain people experienced were unclear. Therefore, it has not yet been determined whether nostalgia changes actual tolerance for painful stimuli. To make a convincing case that nostalgia reduces pain, we conducted Study 2 in a controlled laboratory setting with an algometer, a well-validated measure of pain tolerance that has been utilized in previous research (e.g., DeWall and Baumeister, 2006).

Additionally, although physical pain affects all populations regardless of sociodemographic variables (e.g., age, gender, income, race/ethnicity, geography), a body of work suggests that cultural differences exist with respect to pain tolerance and expression. Even within the United States, the frequency and pain experience vary by ethnicity (Perry et al., 2019; Vaughn et al., 2019).

## Study 2

People from Asian countries (generally) and individuals living in Japan (specifically) report a greater tendency to experience pain than do persons from the United States or Europe (Rowell et al., 2011). Additionally, research shows that Asian individuals have lower pain thresholds and reduced tolerance for heat (Watson et al., 2005; Kim et al., 2017), cold (Hsieh et al., 2010), capsaicin (Gazerani and Arendt-Nielsen, 2005), and pressure pain (Komiyama et al., 2007) compared to non-Hispanic Whites and African Americans. In Japan, persons believe that the expression of pain behaviors (e.g., verbal complaints and facial expressions) are more socially unacceptable compared to Americans (Hobara, 2005). Despite evidence demonstrating that individuals from Asian countries are more sensitive to pain than are those from other societies, research has yet to examine how nostalgia can be utilized to increase capacity for pain among these samples.

The purpose of Study 2 was twofold. First, whereas Study 1 investigated whether nostalgia buffers pain severity for chronic sufferers, Study 2 measured college students’ tolerance for inflicted pain in a laboratory setting. Second, we tested whether nostalgia has the potential to reduce physical pain perceptions among Japanese individuals. To do this, participants were exposed to the same nostalgia manipulation used in the previous experiment (Wildschut et al., 2006). The dependent variable consisted of a pain pressure task where an algometer was placed on the person’s non-dominant hand to assess pain tolerance (DeWall and Baumeister, 2006). It was hypothesized that participants would show greater tolerance for pain following thoughts of nostalgia in comparison to the control condition.

### Method

#### Participants

Utilizing G^∗^Power (Faul et al., 2007) for a mixed-design ANOVA, and based on the effect size from Study 1 (*d* = 0.79, *f* = 0.40) with power set to 0.80, a minimum of 16 persons were needed. We recruited 81 undergraduate students (41 females; *M*_*age*_ = 22.55) from Kyoto University in Japan, who received ¥1000 Japanese Yen (i.e., approximately $8.79 American dollars).

#### Materials and Procedure

Participants completed a “personality and physiological sensation” study in a laboratory setting. After providing informed consent, participants took part in a baseline pain pressure task. Similar to Study 1, participants were randomly assigned to write about a nostalgic or ordinary event, and then all participants completed a final pain pressure task. All study materials took approximately 20 min to complete.

##### Time 1 pain pressure task

Pain tolerance was measured using a pressure algometer (Wagner Instruments FPX 25; Greenwich, CT, United States). This device assesses the amount of pressure applied to a bone or muscle and has been commonly used as a method of assessing pain tolerance in medicine and psychology research (e.g., DeWall and Baumeister, 2006; Lacourt et al., 2012, 2015). The algometer (1 cm^2^ rubber-tipped contact area) was gradually applied with increasing pressure (100 kPa/s) perpendicularly at the first dorsal interosseous muscle of the participant’s non-dominant hand. Participants were instructed to say “stop” when the pain became too uncomfortable to continue. When the individual requested the pressure to cease, the algometer was immediately stopped, with the machine automatically recording the amount of pressure applied prior to cessation. Greater pressure applied indicated higher pain tolerance.

The risks involved with the algometer task were minimal. Participants were informed verbally and in writing (i.e., informed consent) that a hand pressure gauge would be used during the study to elicit pain. The task was completely voluntary and individuals could stop participation at any time without penalty. The experimenter checked on persons’ well-being immediately after the task, and again, at the end of the study (i.e., debriefing). Participants were provided with contact information for researchers in the event that they had further questions/concerns about the experiment.

##### Nostalgia manipulation

Participants were exposed to the same writing task and three-item state nostalgia measure (manipulation check) described in Study 1 (α = 0.95).

##### Time 2 pain pressure task

Following the nostalgia manipulation, pain tolerance scores were measured using the same procedures described above (baseline).

### Results and Discussion

#### Manipulation Check

The results revealed a significant effect of condition, *F*(1,76) = 97.22, *p* ≤ 0.001, *d* = 0.74, with participants exposed to the nostalgia prompt (*M* = 6.55, *SD* = 1.53) reporting higher feelings of state nostalgia compared to persons in the ordinary event condition (*M* = 2.83, *SD* = 1.78).

#### Pain Tolerance

A 2 (between-subjects variable: nostalgia prompt vs. ordinary event prompt) × 2 (within-subjects variable: Time 1 vs. Time 2) mixed-design ANOVA was performed on pain scores. The results revealed a non-significant main effect of nostalgia manipulation, *F*(1,79) = 1.84, *p* = 0.179, *d* = 0.27, and a significant main effect of time, *F*(1,79) = 5.07, *p* = 0.027, *d* = 0.60. Importantly, there was a significant nostalgia × time interaction, *F*(1,79) = 18.65, *p* ≤ 0.001, *d* = 0.99 (see Table 2). Simple main effects revealed that nostalgic participants reported higher tolerance for pain at Time 2 compared to Time 1, *F*(1,79) = 21.32, *p* ≤ 0.001, *d* = 1.00. No difference was found between time points for participants who wrote about an ordinary event, *F*(1,79) = 2.16, *p* = 0.145, *d* = 0.31. Conversely, whereas there was no significant difference between nostalgia and control conditions at Time 1, *F*(1,79) = 0.01, *p* = 0.993, *d* = 0.05, participants who recalled a nostalgic event had higher pain tolerance scores at Time 2 compared to individuals who recalled an ordinary event, *F*(1,79) = 6.12, *p* = 0.016, *d* = 0.69.

**TABLE 2:** Pain tolerance scores as a function of nostalgia condition.

	Time 1		Time 2	
	*M*	*SD*	*M*	*SD*
Nostalgia Condition	12.36	4.86	14.75	5.07
Control Condition	12.35	5.61	11.59	6.34

*Higher scores indicate greater tolerance for pain (Study 2).*

Whereas Study 1 suggests that nostalgia can help reduce the perceived severity of naturally occurring chronic pain, the results of the present experiment demonstrate that students also respond to nostalgic reflection with a heightened tolerance for pain following a mechanically induced pain procedure. Specifically, although there was no significant difference between nostalgia and control conditions on perceptions of pain at baseline, individuals exhibited higher pain tolerance when primed with thoughts of nostalgia versus an ordinary event. An additional benefit of this experiment was to assess the effects of nostalgia on pain using a Japanese sample of participants. Given that pain tolerance can vary as a function of the society in which one resides (Rowell et al., 2011), this study suggests that nostalgia may help both American and Japanese persons. This is especially important as the prevalence of chronic pain disabilities has become a global health priority (Goldberg and McGee, 2011).

## General Discussion

These experiments showed that individuals primed with nostalgic reflection reported a reduction in chronic pain severity (Study 1) and temporary pain tolerance (Study 2). Additionally, whereas Study 1 was conducted among an American sample of participants, Study 2 demonstrated parallel effects in Japanese persons. These findings demonstrate the restorative function of nostalgic reflection in that individuals from two varied cultures reported increased pain resilience in response to nostalgia.

Several studies have examined the restorative function of nostalgic reverie with respect to its capacity to offset discomforting states (e.g., meaninglessness and existential threat) and reinstate psychological equanimity (see e.g., Sedikides and Wildschut, 2020). Related results have been found with respect to temperature-related discomfort (Zhou et al., 2012). Whereas Zhou and colleagues showed that the emotion of nostalgia functions to increase participants’ tolerance for noxious cold, our results found the potential for nostalgia to alleviate physical discomfort more broadly for individuals experiencing chronic and acute pain. This is important, as some studies have criticized the use of cold pressor tasks (used by Zhou et al., 2012), given it is unclear how they are associated with pain experiences in the real world (Birnie et al., 2014).

The current research is also one of a handful of studies to examine the cross-cultural implications of nostalgia. Hepper et al. (2014) research spanned across 18 countries (including American and Japanese participants) and five continents to survey global perceptions of nostalgia. Participants displayed a high level of agreement when defining the features of nostalgia - in particular, *remembering*, *longing/yearning*, and *fondness*. Our Study 2 adds to these findings with a Japanese population, an understudied group in the emotion and nostalgia literatures.

In light of this work, one question is *how* or *why* nostalgia leads to heightened pain resilience. First, prior work has demonstrated that nostalgic reflection increases optimism, self-esteem, positive affect, and perceived social support (see e.g., Sedikides and Wildschut, 2020). These same variables have been found in the health literature to reduce the pain experience (e.g., Affleck et al., 2001; Strand et al., 2006; Sturgeon and Zautra, 2010). Second, nostalgia may play a motivational component, fostering beliefs that one *can* achieve goals when reminiscing about the past (see e.g., Sedikides and Wildschut, 2020). In this way, participants may have responded to the nostalgia prime with a motivation to reframe their painful experiences in a less noxious way. Future research should test these and other possible explanations for the mechanism by which nostalgia reduces pain.

Importantly, we are not recommending nostalgia as an initial treatment or a substitute for other interventions when serious medical attention is required. Our manipulation did not actually treat the source of pain or alleviate an underlying condition; rather, it helped people manage the discomfort they felt. Whereas nostalgic thought may have tangible, physiological outcomes, much more research is needed before making claims about it as a viable treatment option. For instance, although our participants were blind to treatment conditions (i.e., nostalgia vs. control), work should be done wherein individuals are privy to the possibility of nostalgia as a pain reducing intervention. Before practitioners can legitimately recommend or use nostalgia among patients, we need to know how it affects pain resilience when people are aware of its purpose.

The current work is not without limitations. The first experiment was conducted with an MTurk population to extend the generalizability of the results, but it is not known whether individuals were verifiably chronic pain sufferers given an inability to confirm medical diagnoses. Future work should aim to replicate the current findings in a controlled laboratory setting with people clinically diagnosed with chronic pain conditions; an additional benefit of studying this population would be access to a more detailed account of their medical history, related (or not) to current pain problems.

Also, the current research did not assess for health conditions co-existing with chronic pain. The pain experience is often accompanied with other serious health detriments, including mental (e.g., depressive and anxiety) and physical ailments (e.g., hypertension and sleep disturbances). If nostalgia is effective at reducing pain severity for chronic pain sufferers, it may also help reduce comorbid health abnormalities. This is supported by research demonstrating that nostalgia is effective at ameliorating feelings of loneliness (Zhou et al., 2008), lowering stress (Routledge et al., 2011), buffering relational, existential, and death anxieties (e.g., Routledge et al., 2008; Juhl et al., 2010), heightening a sense of vitality (Routledge et al., 2011), and promoting health behaviors (Kersten et al., 2016). Future studies screening pain populations might ascertain the extent to which chronic disabilities are comorbid with other disorders, and how such disorders may impair health.

It is also important to examine whether pre-existing pain severity and/or duration moderate the relationship between nostalgic reflection and pain tolerance. Although the beneficial qualities of nostalgia transcend age, gender, and culture (Sedikides et al., 2015), it is unclear whether these effects would translate to chronic pain sufferers, as the condition is a multidimensional phenomenon that can vary widely (e.g., intensity and persistence). Similar to Verplanken’s (2012) findings that individuals who were high (vs. low) in habitual worrying respond to nostalgic reflection with greater signs of anxiety and depression, it is possible that nostalgia may only be an effective remedy for persons with low or moderate pain severity.

Finally, the current studies focused on the immediate benefits of nostalgia on pain reduction; however, future research should test its capacity to provide more long-term relief. To consider it a legitimate method of treatment for chronic sufferers, it is important to measure the duration of nostalgic thought and to test ways to revisit nostalgia over time to gain the most effective outcomes. Relatedly, a direction for future research is to examine carryover effects from reduced pain following thoughts of nostalgia with respect to psychological, emotional, and social health. Given the detrimental effects of chronic pain on many aspects of life, it would be valuable to test how diminished physical pain could improve individual outcomes, such as mood, life satisfaction, and meaning in life.

Despite limitations, these studies provide initial evidence for nostalgia’s capacity to reduce the severity of physical pain and increase pain tolerance. Given that nostalgia is internally generated, universally experienced, and exempt from serious health costs or risks, it could serve as an easily implemented, widely available, and highly safe alternative pain intervention. This is important given racial, income, and geographic disparities that might limit accessibility to quality healthcare for some individuals (Lynch et al., 2008). For medical professionals looking to recommend immediate forms of pain relief, nostalgia may be useful with other forms of medication and/or mind-body techniques to maximize the relief of chronic physical pain.

In closing, given the prevalence of chronic pain, its effects on well-being, and the difficulty in treatment, it remains important to identify factors that modulate the severity of pain. Similar to other biopsychosocial techniques that are effective therapies for people seeking help with pain, nostalgia may also serve as a promising, cost-effective promoter of homeostasis that is available to all ages, genders, and cultures. To address the silent epidemic of chronic pain conditions worldwide, the present work demonstrated that sufferers may find solace by attenuating pain with the past.

## Data Availability Statement

The raw data supporting the conclusions of this article will be made available by the authors, without undue reservation.

## Ethics Statement

The studies involving human participants were reviewed and approved by the Texas Christian University (TCU) Institutional Review Board (IRB). The patients/participants provided their written informed consent to participate in this study.

## Author Contributions

MK and CC conceived the original project idea. MK, CC, TK, KN, and TW collected the data for all the experiments. MK, JS, and CC analyzed all the data. MK wrote the manuscript with support from JS, CC, and TK. All authors contributed to the article and approved the submitted version.

## Conflict of Interest

The authors declare that the research was conducted in the absence of any commercial or financial relationships that could be construed as a potential conflict of interest.
